# M2 macrophages are closely associated with accelerated clavicle fracture healing in patients with traumatic brain injury: a retrospective cohort study

**DOI:** 10.1186/s13018-018-0926-7

**Published:** 2018-08-29

**Authors:** Ran Zhang, Yi Liang, Shuxiang Wei

**Affiliations:** 1 Department of Orthopedics, Liuzhou General Hospital, 8 Wenchang Rd, Liuzhou, 545006 Guangxi China; 2 Guangxi University of Technology, Liuzhou, 545006 Guangxi China

**Keywords:** Traumatic brain injury, Clavicle fracture, Fracture healing, Macrophages

## Abstract

**Background:**

Mounting evidence indicate patients with traumatic brain injury (TBI) have an accelerated fracture healing. The healing process of bone fractures is greatly dependent on infiltrated macrophages. The macrophages are categorized into M1 or M2 phenotypes with different functions. This study is aimed to address the potential role of subtypes of macrophages in the process of fracture healing in patients with TBI.

**Methods:**

Twenty-five cases of clavicle fracture alone (CF group) and 22 cases of clavicle fracture concomitant with TBI (CFT group) were retrospectively analyzed in this study. Callus tissues were harvested during operations. The expressions of COX-2, CD206, and CD68 were measured with immunohistochemistry.

**Results:**

The percentages of M2 macrophages in total macrophages increased after bone fracture in both groups, while the percentages of M1-type macrophages are decreased. Interestingly, the increased percentages of M2 macrophages are significantly higher in CFT group than in CF group. Compared to CF group, the fracture callus volume was much larger (21.9 vs 8.5 cm^3^) and the fracture healing time was much shorter (82.2 vs 127.0 days) in CFT group. The percentage of M2 macrophages was negatively correlated with fracture healing time in patients (*r* = − 0.575, *p* < 0.01).

**Conclusions:**

The findings suggest that the percentages of M2 macrophages in callus tissues increased dramatically during the repairing stage in both CF and CFT group. Percentages of M2 macrophages are associated with accelerated fracture healing in patients with TBI. M2 macrophage polarization during the stage of bone regeneration may play a vital role in promoting bone fracture healing.

## Background

Traumatic brain injury (TBI) is one of the most severe trauma-induced injuries and is one of the leading cause of death and disability. TBI is usually accompanied with peripheral bone fractures. The interaction of TBI and concomitant fracture has been under long-term discussion [1, 2]. In the last decade, multiple clinical and preclinical studies have shown an association between TBI and accelerated fracture healing [3–7]. However, the mechanism underlying this relationship needs to be further studied.

Fracture healing is a sophisticated biological process involving numerous types of cells and signals. After the bone fracture, immune cells are rapidly recruited to the damage site and secrete inflammatory factors. This intense inflammatory response is required to facilitate the recruitment and activation of hematopoietic and mesenchymal cells, which promote normal fracture healing through the angiogenesis and regeneration of the injured tissue [8]. Recently, macrophages, phagocytic cells of the myeloid lineage, have been uncovered to play an important role in bone fracture healing [9, 10]. Macrophages can be arbitrarily classified into two groups: classically activated (M1-type) macrophages and alternatively activated (M2-type) macrophages [11]. M1-type macrophages secrete interleukin-1 (IL-1), interleukin-6 (IL-6), tumor necrosis factor alpha (TNF-α), monocyte chemoattractant protein 1, and macrophage inflammatory protein 1 to initiate an inflammatory response in the early stages of inflammation, thus recruiting monocyte to remove necrotic cells and the fibrin thrombus [12]. After inflammation response, M2-type macrophages were recruited to the damage site and secrete tissue repair signals, such as interleukin-10 (IL-10), transforming growth factor beta (TGF-β), bone morphogenetic protein 2, and vascular endothelial growth factor, to initiate an anti-inflammatory response in the later stages of inflammation as well as recruit mesenchymal progenitor cells, induce osteochondral differentiation, and enhance angiogenesis [13–16].

Although investigations concerning the correlation of macrophages and fracture healing have been extensively studied [17–19], to our knowledge, there has been no study on the association of macrophage subtypes with accelerated fracture healing in TBI patients so far. Therefore, the present study aimed to address the potential role of both M1 and M2 types of macrophages in the process of accelerated fracture healing in patients with both bone fracture and traumatic brain injury.

## Methods

### Study subjects

This study was conducted under the guide of the Declaration of Helsinki and approved by the Ethical Review Committee of the Liuzhou General Hospital. Written informed consent was obtained from all subjects prior to their participation in the study. Forty-seven patients with clavicle fracture were admitted to the Department of Orthopedics, Liuzhou General Hospital, between January 2009 and July 2017. The fractures in all patients were treated operatively. Twenty-five patients with clavicle fracture alone (CF group) underwent open reduction and internal fixation of the clavicle within 1 or 2 weeks after injury. Twenty-two patients with clavicle fracture and concomitant TBI (CFT group) consisted of 20 cases caused by traffic accidents and 2 cases caused by fall injury. Among these 22 patients, 12 patients with TBI were treated with open reduction and internal fixation of the clavicle within 1 week post-injury and 10 patients underwent surgery between 7 and 14 days after brain injury. After admission, the patients of two groups were both examined by conventional X-rays for the clavicle fracture and evaluated with the Glasgow Coma Scale (GCS) system. Callus volume measurement of bone fracture before operation, length, width, and height, was obtained from computed tomography (CT) scan images before surgery. We use the following formula to calculate the callus volume: volume = length × width × height × π/6, and the width and height of callus tissue in the formula were subtracted the width and height of clavicle bone respectively. The CFT group was diagnosed with computerized tomography of the head and the GCS score is less than 8 points, while the CF group without TBI, the GCS score is more than 13 points. Participants with any forms of prior nervous system or bone-related diseases, multiple fractures, autoimmune disease, malignant disease, diabetes, rheumatoid arthritis, or other chronic inflammation diseases as well as history of long time steroid, non-steroidal anti-inflammatory drugs, and immunosuppressant or bisphosphonate therapy or survived less than 1 year after surgery were excluded.

### Follow-up and assessment of fracture healing

All patients were followed up for at least 24 weeks after the operation. The follow-up examination was based on clinical and radiological examination at 1, 2, 4, 8, 12, 16, 20, and 24 weeks after trauma. The fracture was recorded as clinical heal based on the following clinical indications: (1) the patient had no pain at the surgical site and no tenderness; (2) the affected side could lift a 1-kg weight for 1 min; and (3) the X-ray photographs showed that the fracture line was blurred and there was a continuous epiphyseal fracture line. Two radiologists, who were blinded to the presence or absence of TBI, independently evaluated the bone healing.

### Immunohistochemistry

During the operation, the callus tissue between fractures was examined by immunohistochemistry with anti-CD68 (MXB Biotechnology, cat#: Kit-0026), anti-COX-2 (MXB Biotechnology, cat#: RMA-0549), and anti-CD206 (Santa Cruz Biotechnology, cat#: sc-376108). After antigen repair, H_2_O_2_ was added to the section and incubated for 10 min at room temperature to block the endogenous peroxidase; the PBS buffer was used to wash for 3 min × 3 times. One hundred microliters of the blocking normal calf serum working solution was added and incubated for 15 min at room temperature. After removing the serum, add 100 μl of primary antibody and store overnight at 4 °C. After warming at room temperature for 15 min on the second day, the PBS buffer was used to wash for 3 min × 3 times. One hundred microliters or an appropriate amount of biotin-labeled goat anti-mouse/rabbit IgG polymer was added and incubated at room temperature for 13 min; phosphate-buffered saline (PBS) buffer was used to wash for 3 min × 3 times. Add 100 μl or an appropriate amount of horseradish peroxidase-labeled streptolysin working solution and incubate at room temperature for 10 min; rinse with PBS buffer for 3 min × 3 times. An appropriate amount of freshly prepared DAB was added and incubated for 5–8 min. Counterstaining was performed using tap water, and hematoxylin stain was incubated for 20 s; differentiation and flushing returned to blue.

### Image analysis

Immunohistochemistry was performed on each slice, and five fields of view were selected from the top left, top right, bottom right, bottom left, and the middle and were imaged with the Minmei microscope digital imaging system (V9.5.2). The images were analyzed with Image-pro plus 6.0 (Media Cybernetics, Rockville, MD, USA). The immunohistochemistry images were opened in Image-pro plus software. Using count and measure functions, positive cells were manually selected in several images in order to train the software. After that, all positive cells were automatically counted and processed by the software while the sensitivity was set to level 4.

### Statistical analysis

Statistical analysis was performed with the Statistical Package for Social Sciences software (SPSS Inc.; Chicago, IL, USA), version 16.0 for Windows. All data were presented as mean, standard deviation of the mean (SD), or median (interquartile range). Kolmogorov-Smirnov test was performed to analyze the data normality, and Student’s *t* test, Mann-Whitney *U* test, or Chi-square test were used to assess the significance in clinical characteristics between patients with fracture only and concomitant TBI. Pearson correlation was used to assess significance among healing time and clinical variables. *p* < 0.05 was considered to be statistically significant for differences and correlations.

## Result

### Baseline characteristics of the study groups

**Fig. 1:**
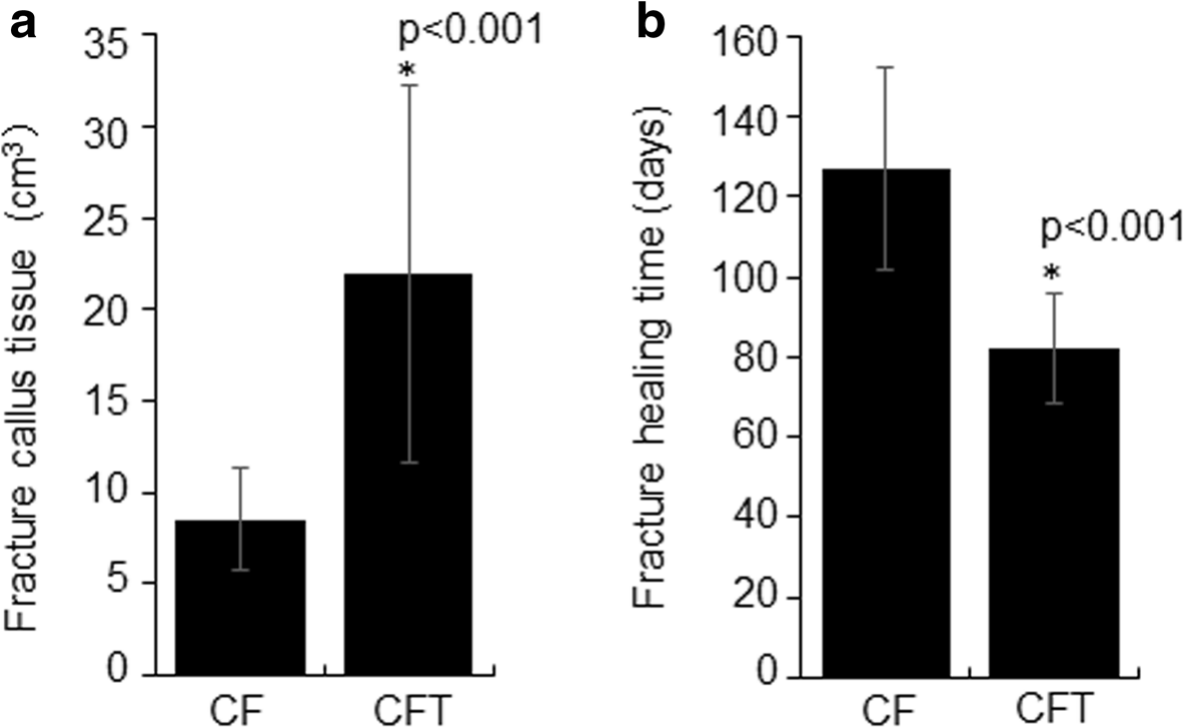
Fracture callus volume and healing time in clavicle fracture alone (CF group) and clavicle fracture concomitant TBI (CFT group). *: the difference is statistical signficance between the CF and CFT groups

**Fig. 2:**
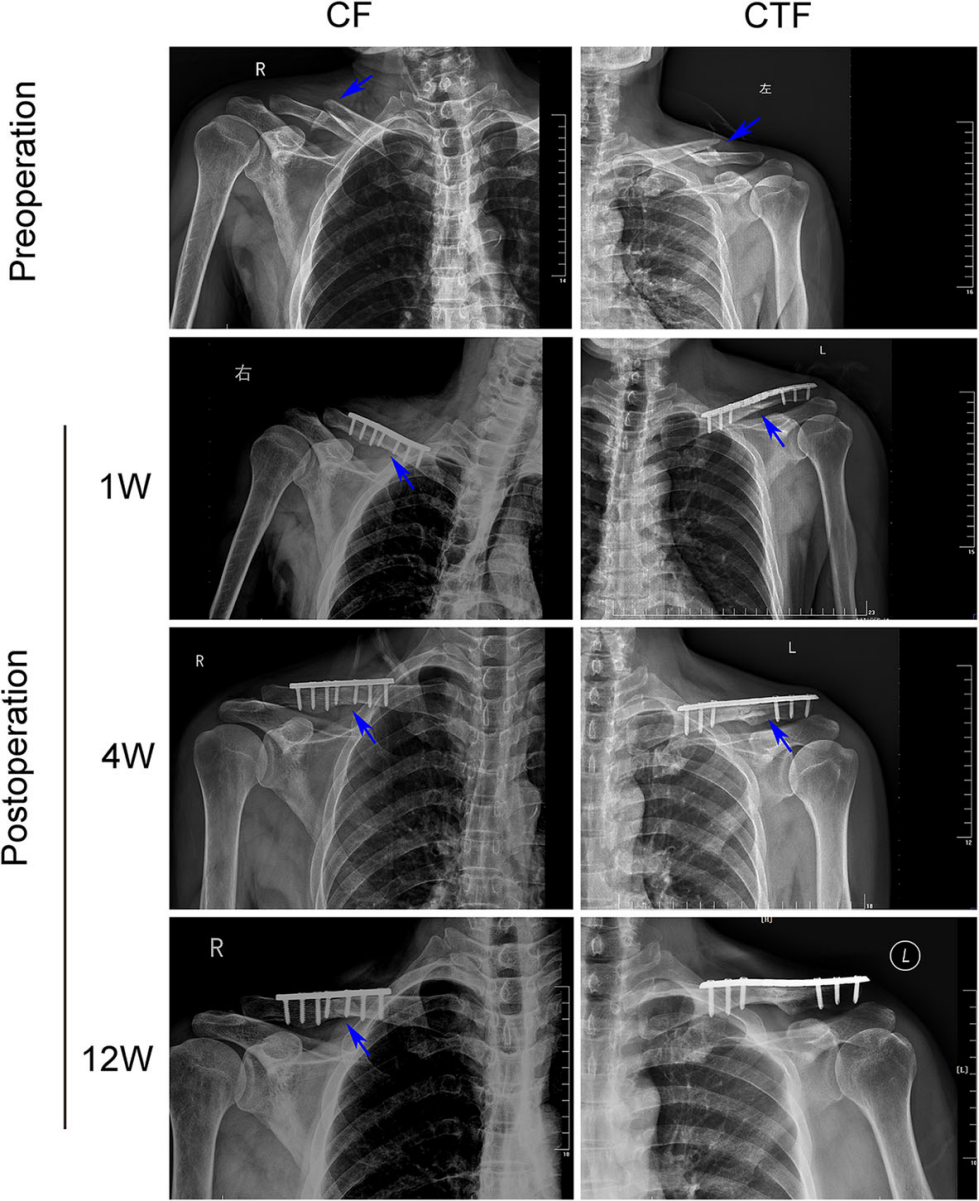
The representative chest radiograph of the clavicle fracture alone (CF group, left panels) and clavicle fracture concomitant TBI (CFT or called CTF group, right panels) showed fixation at around 1 week, 4 weeks, and 12 weeks after surgery. Healed clavicle fracture was seen at 12 weeks after surgery in the patient of CFT (CTF) group, but not in the CF patient. The arrows pointed the fracture lines

Total of 25 patients with fracture alone (CF group) and 22 patients with fracture and concomitant TBI (CFT group) were included in this study. The demographics and clinical profile of patients in CF and CFT groups are summarized in Table 1. There are no significant differences in baseline clinical parameters, including age, gender, fracture place, and time from injury to surgery between the two groups (for all, *p* > 0.05). The CFT group has significantly lower GCS scores (11.5 vs 15,) and blood monocyte (0.058 vs 0.922) (Table 1), larger fracture callus volume (21.9 vs 8.5 cm^3^) (Fig. 1a), and shorter healing time (82.2 vs 127.0 days) (Fig. 1b) compared with the CF group (for all, *p* < 0.001). Callus volume is a very important indicator of bone fracture healing. If the other indicators are similar, the larger the size of the callus at the injury site, the faster the fracture healing. The preoperative photographs of X-ray showed the healing process of clavicle fractures in both groups, and the photographs of X-ray at 12 weeks after surgery showed that the fracture recovery was much faster in the CFT group than in the CF group (Fig. 2).

**Table 1:** The demographics of clinical profile of patients in CF and CFT groups

Features	CF group	CFT group	*p* value
Number	25	22	–
Age (years)	36.0 ± 15.3	34.0 ± 15.2	0.657
Gender			0.820*
Male	21 (84.0)	19 (86.4)	
Female	4 (16.0)	3 (15.6)	
GCS score	15 ± 0	11.5 ± 1.7383	0.000
Blood monocyte (%)	0.922 ± 0.020	0.058 ± 0.035	0.000
Time from injury to surgery			0.706*
≤ 7 days	10 (40.0)	10 (45.5%)	
8–14 days	15 (60.0)	12 (54.5%)	
COX-2/CD68 (%)	29.95 ± 11.90	33.54 ± 9.3	0.259
CD206/CD68 (%)	20.23 ± 8.08	40.53 ± 18.16	0.000

Data were shown in mean (STD) or *n*/*N* (%). Student’s *t* test was used in all other analysis. *GCS* Glasgow Coma Score

*Chi-square test was used to the analysis

### The percentage of M2-type macrophages increased during repairing stage

**Fig. 3:**
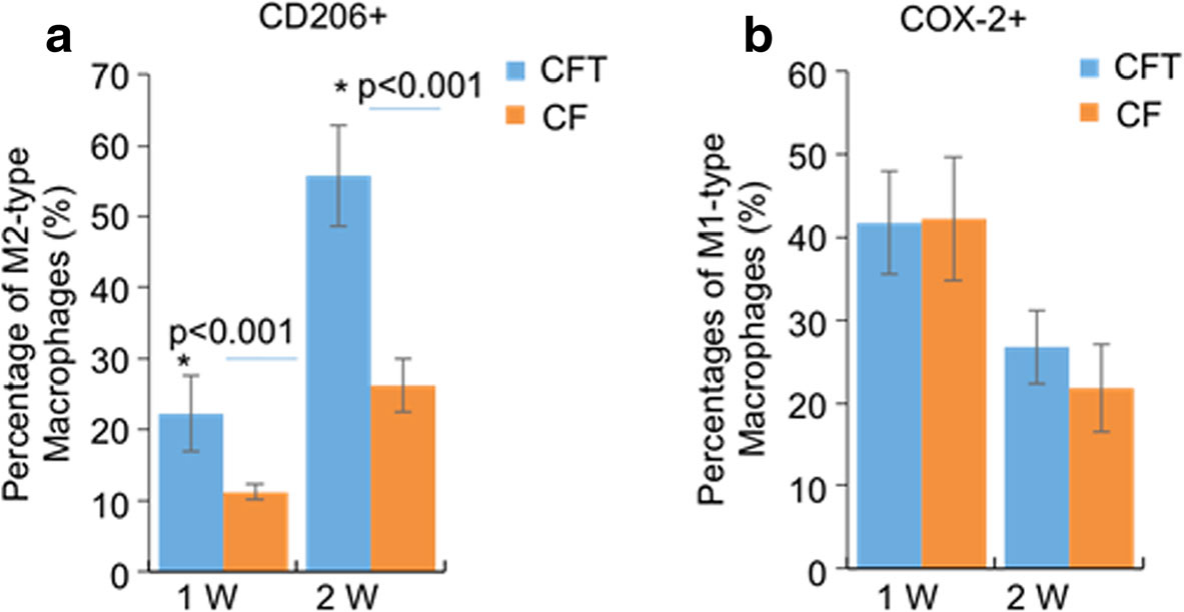
The percentages of M1-type macrophage, COX-2 positive macrophages and the percentages of M2-type macrophages, CD206 positive macrophages in all macrophages (CD68 positive macrophages) in clavicle fracture alone (CF group), and clavicle fracture concomitant TBI (CFT group). The percentages of M1-type macrophage did not show significant difference between the CF and CFT groups of patients who underwent surgery within 1 week or between 8 days and 14 days after injury. The percentages of M1-type macrophage showed the decrement trend with time following the fracture in patients with and without traumatic brain injury. *: the difference is statistical significance between the CF and CFT groups

**Fig. 4:**
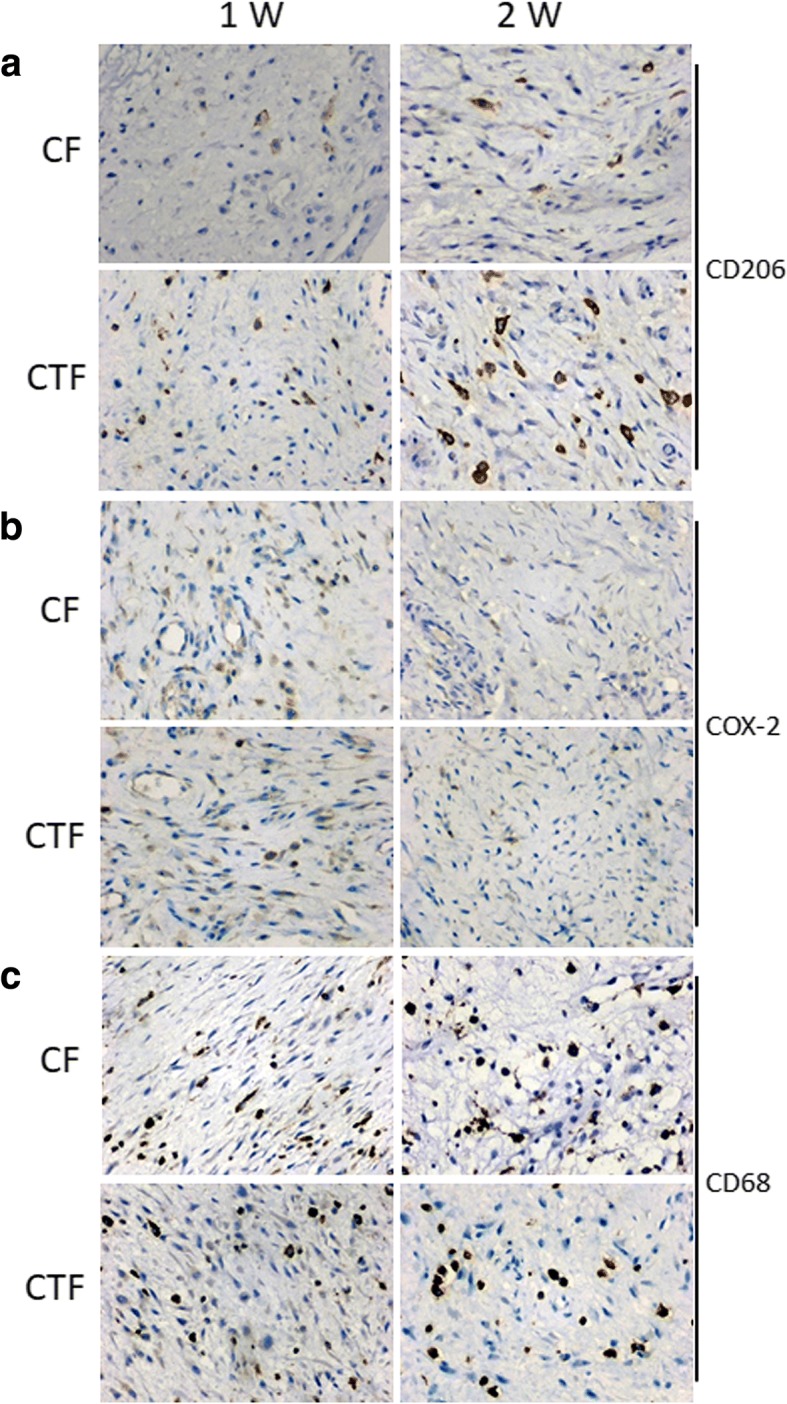
The representative immunohistochemistry images of the expression of COX-2, CD206, and CD68 in clavicle fracture alone (CF group) and clavicle fracture concomitant TBI (CFT or called CTF group). (Scale bars: 50 μm)

To characterize the relationship between macrophages and fracture healing, we measured the expression of COX-2 (M1-type macrophage marker), CD206 (M2-type macrophage marker), and CD68 (all macrophages marker) in the callus tissues after bone fracture for 1 week and 2 weeks with immunohistochemistry. Each section was randomly chosen five fields to be photographed, and the images were analyzed with Image-pro plus software. The percentages of M1-type macrophages decreased after bone fracture for 1 week and 2 weeks in both the CF group and CFT group (*p* < 0.001) (Figs. 3a and 4a), while the percentages of M2-type macrophages increased over time (Figs. 3b and 4b).

### The percentage of M2-type macrophages elevated in patients with clavicle fracture and concomitant TBI

**Fig. 5:**
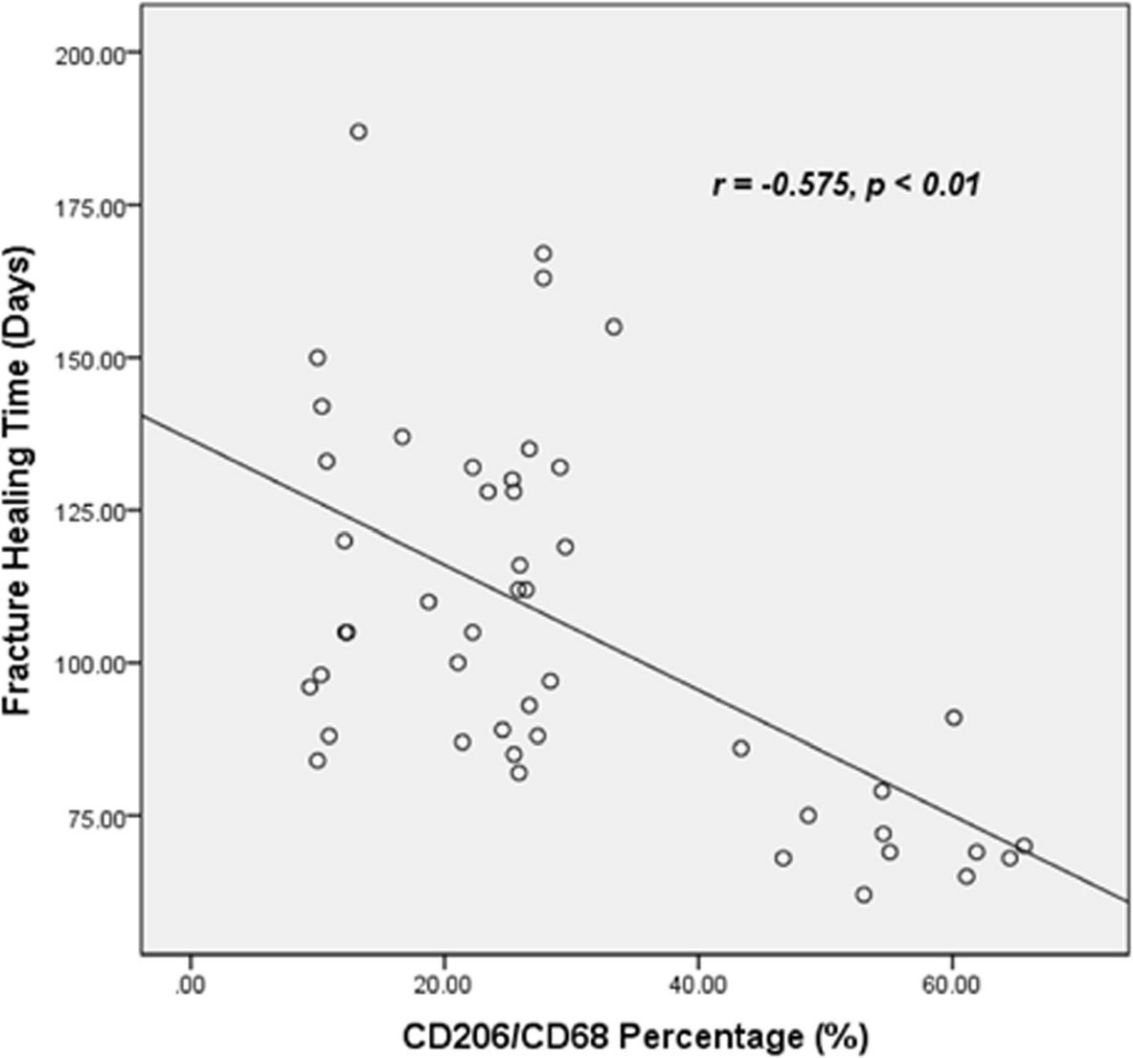
The correlation between fracture healing time and the percentage of M2-type macrophages

The percentages of M1-type macrophage, COX-2 positive macrophages, did not show significant difference between the CF and CFT groups of patients who underwent surgery within 1 week or between 8 and 14 days after injury (*p* > 0.05) (Figs. 3a and 4a), but the percentages of M1-type macrophage in both CF and CFT groups of patients who underwent surgery between 8 and 14 days after injury were significantly decreased, compared to that of patients who underwent surgery within 1 week after injury. The percentages of M2-type macrophages are significantly increased in both the CF group and CFT group of patients with time before surgery. Meanwhile, the increase of percentages of M2-type macrophages was significantly higher in the CFT group of patients than that in the CF group of patients who took surgery within 1 week (22.27% vs 11.21%, *p* < 0.001) and between 8 and 14 days (55.75% vs 26.25%, *p* < 0.001) after injury, respectively (Figs. 3b and 4b). In addition, the CD68 positive macrophages are not significantly different from these two groups over time (Fig. 4c). There is a significant negative correlation between fracture healing time and the percentage of M2-type macrophages in callus tissues (*r* = − 0.575, *p* < 0.01) (Fig. 5 and Table 2). The fracture healing time is also shown to be positively correlated with GCS scores and monocyte percentage in blood and negatively correlated with the callus volume (Table 2, *p* < 0.01). but not the percentage of M1-type macrophages, age, gender, fracture place, and time from injury to surgery (Table 2, *p* > 0.05). These results suggest that M2-type macrophages are more activated and recruited to the injury sites in patients with clavicle fracture and concomitant TBI.

**Table 2:** Pearson correlations among healing time and clinical variables

		Healing time	Age	Gender	GCS	Injury type	Surgery time after injury	Callus volume	COX2	CD206	Monocyte percentage
Healing time	r	1	0.110	− 0.051	0.746**	− 0.744**	− 0.037	− 0.627**	− 0.192	− 0.575**	0.359*
	p		0.464	0.733	0.000	0.000	0.803	0.000	0.196	0.000	0.013
Age	r	0.110	1	0.049	0.084	− 0.066	0.005	0.043	0.113	− 0.116	0.090
	p	0.464		0.744	0.575	0.657	0.971	0.773	0.450	0.436	0.547
Gender	r	− 0.051	0.049	1	− 0.074	0.033	0.123	0.097	− 0.048	0.117	0.097
	p	0.733	0.744		0.622	0.825	0.408	0.518	0.749	0.433	0.515
GCS	r	0.746	0.084	− 0.074	1	− 0.836	− 0.268	− 0.826	0.103	− 0.785	0.478
	p	0.000	0.575	0.622		0.000	0.068	0.000	0.489	0.000	0.001
Injury type	r	− 0.744	− 0.066	0.033	− 0.836	1	− 0.055	0.683	0.168	0.602	− 0.520
	p	0.000	0.657	0.825	0.000		0.713	0.000	0.259	0.000	0.000
Time from injury to surgery	r	− 0.037	0.005	0.123	− 0.268	− 0.055	1	0.194	− 0.834	0.665	0.073
	p	0.803	0.971	0.408	0.068	0.713		0.191	0.000	0.000	0.625
Callus volume	r	− 0.627	0.043	0.097	− 0.826	0.683	0.194	1	0.001	0.720	− 0.311
	p	0.000	0.773	0.518	0.000	0.000	0.191		0.995	0.000	0.033
COX2	r	− 0.192	0.113	− 0.048	0.103	0.168	− 0.834	0.001	1	− 0.452	− 0.076
	p	0.196	0.450	0.749	0.489	0.259	0.000	0.995		0.001	0.612
CD206	r	− 0.575	− 0.116	0.117	− 0.785	0.602	0.665	0.720	− 0.452	1	− 0.212
	p	0.000	0.436	0.433	0.000	0.000	0.000	0.000	0.001		0.152
Monocyte percentage	r	0.359	0.090	0.097	0.478	− 0.520	0.073	− 0.311	− 0.076	− 0.212	1
	p	0.013	0.547	0.515	0.001	0.000	0.625	0.033	0.612	0.152	

**Correlation is significant at the 0.01 level (2-tailed)

*Correlation is significant at the 0.05 level (2-tailed)

## Discussion

In fracture healing, immune system closely interacts with skeletal system through various cell mediators and molecular precursors. Although the whole process of fracture healing is complicated, it is intriguing that macrophages have been reported to play a key role during all stages of fracture healing [20]: M1-type macrophages represent one of the earliest cells recruited into the injury site triggered by tissue injury and thus are considered as part of the acute phase response [21, 22]; M2-type macrophages initiate an anti-inflammatory response in the later stages of inflammation and are essential for tissue remodeling [23–25]. However, the relative importance of M1-type and M2-type macrophages in the subsequent phases of bone regeneration remains poorly understood.

In the present study, we analyzed the percentages of M1-type and M2-type macrophages in the callus tissues of patients with Clavicle fracture alone (CF group) and patients with Clavicle fracture concomitant TBI (CFT group). The percentages of M1-type macrophage showed the decrement trend with time following the fracture in patients with and without traumatic brain injury. Besides the confirmation of previous report that TBI could accelerate the fracture healing [6, 26–28], the percentages of M2-type macrophages increased dramatically with time in both the CF and CFT group, concomitant with a larger fracture callus volume, and the percentages of M2-type macrophages in CFT group were significantly higher than CF group. This agreed with the process of wound healing—first inflammatory stage and then repairing stage with clear angiogenesis after the acute response. It has been reported that a prolonged infiltration of pro-inflammatory macrophages are positively related with unsuccessful bone healing [24, 29]. Our study suggests that M1 macrophages mainly present in the early stage of bone healing process and decrease in the later stage to prevent a prolonged pro-inflammatory. The pro-inflammatory cytokines produced by M1-type macrophages, such as TNFα and IL-6, are known to suppress osteoblast differentiation, thus inhibiting collagen production of osteoblasts and mineralization, while M2-type macrophages express TGFβ and IL-10, which supports bone deposition [30, 31]. The M1:M2 ratio shifts towards M2 macrophage polarization during the stage of bone regeneration may improve the healing outcome by preventing delayed bone regeneration.

To conclude, this study is the first to report that the percentages of M2-type macrophages increased dramatically during repairing stage in both the CF and CFT group, concomitant with a larger fracture callus volume and, eventually, a shorter fracture healing time. Particularly, the percentage of M2-type macrophages elevated in patients with clavicle fracture and concomitant TBI compared with the patients with clavicle fracture alone. However, the present study had several limitations. Although 25 patients with Clavicle fracture alone and 22 patients with Clavicle fracture concomitant TBI were enrolled for pathogenesis analyses, the power to elucidate association between the percentages of M2-type macrophages and clavicle fracture concomitant TBI was limited by small number of subjects. Another important limitation was that animal model was not used to validate the role of M2-type macrophages in fracture concomitant TBI. Therefore, it will be of future interest to study the detailed molecular mechanism of the activation of M2-type macrophages in patients with clavicle fracture concomitant TBI and perform prospective studies based on the results from large samples.

## Conclusions

This study suggests that the percentages of M2-type macrophages increased dramatically during repairing stage in both the CF and CFT group, concomitant with a larger fracture callus volume and, eventually, a shorter fracture healing time. To our knowledge, this is the first report that M2-type macrophages are associated with accelerated fracture healing in patients with TBI. Although we found an association between the percentages of M2-type macrophages and clavicle fracture concomitant TBI with 47 samples, prospective studies are required based on the results from larger samples.

## Abbreviations

CD206: Cluster of differentiation 206
CD68: Cluster of differentiation 68
CF: Clavicle fracture
COX-2: Cyclooxygenase 2
CT: Computed tomography
CTF: Clavicle fracture concomitant TBI
GCS: Glasgow Coma Scale
IL-1: Interleukin-1
IL-10: Interleukin-10
IL-6: Interleukin-6
PBS: Phosphate-buffered saline
TBI: Traumatic brain injury
TGF-β: Transforming growth factor beta
TNF-α: Tumor necrosis factor alpha
